# Crustaceans as Key Prey: Insights Into the Dietary Partitioning of Four Carnivorous Fishes in the Nansha Islands, South China Sea

**DOI:** 10.1002/ece3.71497

**Published:** 2025-06-04

**Authors:** Chen Zhang, Simin Hu, Xianzhi Lin, Hui Huang, Sheng Liu

**Affiliations:** ^1^ Key Laboratory of Tropical Marine Bio‐Resources and Ecology, Guangdong Provincial Key Laboratory of Applied Marine Biology, South China Sea Institute of Oceanology Chinese Academy of Sciences Guangzhou China; ^2^ University of Chinese Academy of Sciences Beijing China; ^3^ Sanya Joint Laboratory of Marine Science Research, Key Laboratory of Tropical Marine Biotechnology of Hainan Province Sanya Institute of Ocean Eco‐Environmental Engineering Sanya China; ^4^ Sanya National Marine Ecosystem Research Station, Tropical Marine Biological Research Station in Hainan Chinese Academy of Sciences Sanya China

**Keywords:** carnivorous fish, coral reef, crustaceans, dietary partitioning, prey diversity

## Abstract

Small carnivorous fishes serve as important mesopredators in coral reef ecosystems. However, the habitat and prey availability degradation within these ecosystems has intensified the trend of body size miniaturization and interspecific competition among these species. To better understand the food selection and resource partitioning strategies of mesopredators, we conducted a comprehensive study on the feeding habits of four small carnivorous fish species collected from the coral reefs of the Nansha Islands. This study employed a combination of morphological analysis and molecular identification of gut contents, along with stable isotope analysis. Similar food items, mostly semi‐digested/undigested body remains/fragments from crustaceans, fish, and mollusk were detected in the guts of the analyzed fishes. High‐throughput sequencing based on DNA barcoding identified approximately 24 taxa belonging to Arthropoda, Chordata, and Mollusca, with Arthropoda being the most abundant prey group, accounting for 82.2%–92% of the total sequences across the four fish species. Stable isotope analysis further revealed that the trophic levels of the four species ranged from 3.4 to 3.6. The results of food overlap analysis based on stable isotopes contrasted with those obtained from high‐throughput sequencing, highlighting the distinct characteristics and complementary strengths of these methods. This study broadens the current understanding of the feeding ecology of four carnivorous fish species. The findings reveal that crustaceans are the primary food source for carnivorous fishes in the Nansha Islands, differing from previous assumptions that their diets were predominantly fish‐based. Additionally, the differentiated utilization of crustacean resources among these species suggests that marine benthic invertebrates may play a crucial role in supporting mesopredators within degraded coral reef ecosystems, potentially helping to mitigate the environmental stress they face.

## Graphical abstract

This study, through a combination of morphological analysis, molecular identification, and stable isotope analysis, confirmed that crustaceans are the primary food source for these fishes. Small carnivorous fishes reduce niche overlap by selectively feeding on different types of crustaceans—a strategy supported by the abundant crustacean resources in coral reef areas.

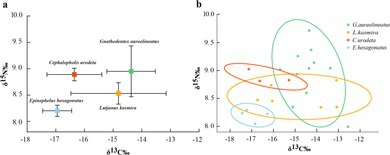

## 1 Introduction

Carnivorous fishes play a pivotal role as top predators within coral reef ecosystems. By preying on animals across multiple trophic levels, including both fish and invertebrates, they exert strong top‐down control on prey populations and indirectly influence primary producers through trophic cascades (Pace 2013). The remarkable diversity of carnivorous fishes is likely driven by the complex structural habitats provided by coral reefs, which not only sustain high biodiversity and abundant food resources but also offer critical opportunities for predator avoidance and ambush hunting (Roberts and Ormond 1987; Messmer et al. 2011; Komyakova et al. 2013; Strona et al. 2021; Kramer et al. 2014, 2015, 2017; Graham and Nash 2013; Kochan et al. 2023; Mills et al. 2023). However, coral bleaching and mortality driven by ocean warming simplify the three‐dimensional structure of coral reefs, diminishing the predation efficiency of carnivorous fishes (Wilson et al. 2008). Additionally, elevated thermal stress significantly reduces fish biomass, with each unit increase in degree heating weeks (DHW) leading to an approximate 12% decline in reef fish biomass and over 20% declines in carnivorous species, particularly invertivores (Cook et al. 2022).

The decline in habitat complexity and food availability intensifies competition among coral reef fishes (Almany 2004; Ward et al. 2006; Messmer et al. 2011; Darling et al. 2017; Dias et al. 2022). However, dietary differentiation among species plays a crucial role in alleviating competition through resource partitioning, ultimately supporting species coexistence. For example, a study of four sympatric cryptobenthic fish species in the coral reef ecosystems of Belize revealed significant dietary divergence. DNA barcoding of stomach contents uncovered notable variations in prey types and abundances among these species, highlighting the role of dietary differentiation in reducing interspecific competition (Brandl et al. 2020). Similarly, research on planktivorous fishes demonstrated that species partition food resources by selectively feeding on distinct plankton types. This selective foraging behavior not only minimizes competition but also promotes the coexistence of diverse species within coral reef communities (Leray et al. 2019).

Early foundational studies have long emphasized the importance of predation and resource availability in shaping coral reef fish communities (Hiatt and Strasburg 1960; Hixon and Beets 1993). These studies highlighted that predator–prey interactions and access to refuges significantly determine the composition and structure of reef assemblages. Building on this foundation, recent work has focused on how environmental degradation may alter these dynamics by impacting food webs and trophic linkages. Coral reef habitats—including living and dead coral, rubble, the epilithic algal matrix (EAM), and sand—support rich and diverse invertebrate assemblages that serve as essential food sources for carnivorous fishes (Head et al. 2018). Among these, crustaceans are especially important, comprising about 20% of invertebrate biomass and acting as key intermediaries in detrital and microbial food webs (Enochs and Manzello 2012; Kramer et al. 2014). These prey resources not only support energy transfer from benthic substrates to higher trophic levels but also offer insight into how carnivorous fishes interact with their environment and one another. Carnivorous fishes exert a strong influence on coral reef ecosystems by regulating prey populations (Tonn et al. 1992; Stewart and Jones 2001) and driving trophic cascades (Mumby et al. 2006, 2012), thereby shaping both community structure and ecosystem function. Therefore, systematically investigating their feeding strategies and ecological adaptability is essential not only for deepening our understanding of the complexity of coral reef food webs but also for providing a theoretical basis for effective conservation and ecosystem management.

Studies of fish diet composition commonly employ gut content analysis and stable isotope analysis, which provide complementary perspectives on feeding ecology. Gut content analysis offers high taxonomic resolution and reflects recent feeding events, making it a standard tool for identifying prey items. Morphological identification, traditionally used in this approach, is straightforward and cost‐effective, and allows for the direct observation of recognizable prey remains (Choat et al. 2002; Wu et al. 2022). Nonetheless, when prey is highly digested or lacks distinct features, this method can underestimate dietary diversity or obscure nutritional patterns. Stable isotope analysis, in contrast, is widely used to examine trophic position and ecological niche at the community level. δ^15^N is commonly used to indicate an organism's trophic level, while δ^13^C reflects the basal sources of primary production in the food chain (Minagawa et al. 1984; Zanden and Rasmussen 2001; Post 2002; McCutchan et al. 2003). It enables researchers to quantify isotopic niche width and resource overlap, offering functional insights into trophic relationships that complement the taxonomic focus of gut content analysis (Wyatt et al. 2012; Yin et al. 2023). High‐throughput sequencing (HTS) has more recently emerged as a powerful molecular approach in diet studies. It facilitates the detection of degraded or morphologically indistinct prey DNA, substantially improving taxonomic resolution. HTS has been applied successfully to various marine taxa, including copepods (Hu et al. 2015), chaetognaths (Wang and Hu 2020), spotted scat ( *Scatophagus argus* ) (Lin et al. 2018), and orange‐lined triggerfish ( *Balistapus undulatus* ) (Zhang and Hu 2022). While this method offers fine‐scale dietary detail, it captures only a temporal snapshot of feeding and may be affected by factors such as DNA degradation, sample preservation, and amplification bias (Pompanon and Deagle 2012; Deagle et al. 2013, 2019). The integration of gut content analysis, stable isotope analysis, and HTS provides a more comprehensive understanding of fish feeding ecology. Each method contributes unique strengths across temporal, functional, and taxonomic dimensions, and their combined application can better resolve trophic structure and resource use in marine ecosystems.

The South China Sea, located in the Indo‐Malaysian center of the Indo‐West Pacific region, features diverse and complex habitats, making it one of the most biodiverse tropical regions in the world (Huanting and Lirong 2017). In this study, we comprehensively investigated the dietary habits of four carnivorous coral reef fish using an integrated approach that includes gut content observation, stable isotope analysis, and cytochrome c oxidase subunit I (COI) HTS. The focus was on comparing the feeding selectivity of these carnivorous coral reef fish, including striped large‐eye bream ( *Gnathodentex aureolineatus* ), common bluestripe snapper ( *Lutjanus kasmira* ), darkfin hind ( *Cephalopholis urodeta* ), and starspotted grouper ( *Epinephelus hexagonatus* ). These species are typical carnivorous fishes in the coral reef ecosystems and are also mesopredators of the coral reef food web (Froese and Pauly 2024; Li and Zhang 2020; Shao 2024; Zhang and Chen 2015). This study aimed to analyze the diets of carnivorous fish using a combination of methodological approaches, leveraging the strengths of each technique to offset their respective limitations. Additionally, we aimed to investigate how these fish achieve resource partitioning and coexistence under habitat degradation by examining dietary differentiation based on high‐resolution dietary composition data.

## 2 Methods

### 2.1 Study Region and Survey Methods

**FIGURE 1:**
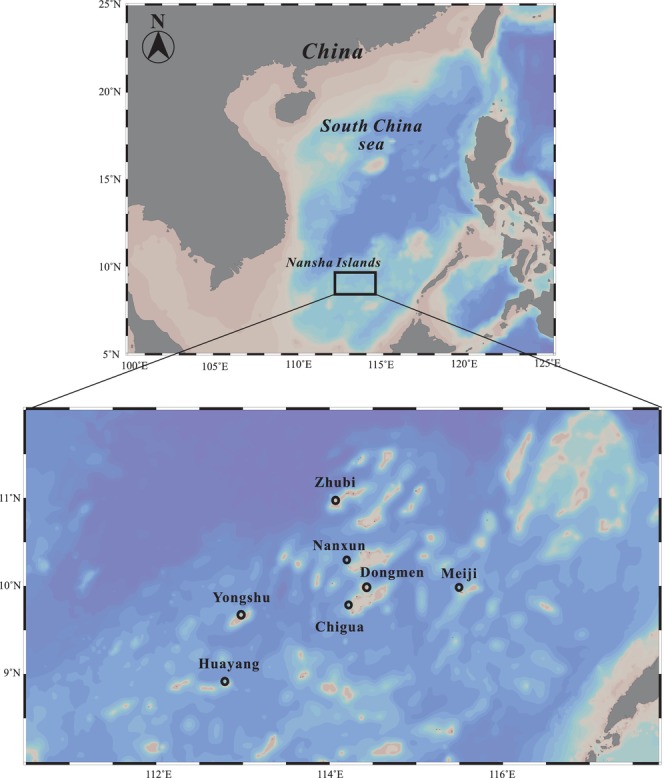
The study area is located in the South China Sea, and the distribution of research stations is marked. The upper map illustrates the broad geographical location of the South China Sea, with the specific location of the Nansha Islands marked. The lower map provides a zoomed‐in view of the Nansha Islands, highlighting specific sampling stations, including Zhubi, Nanxun, Dongmen, Chigua, Meiji, Yongshu, and Huayang.

A survey and sampling study was conducted in seven reefs within the Nansha Islands. The study area is shown in Figure 1, and the research was conducted from spring 2016 to spring 2017. The study station map was created using Ocean Data View (Version 5.6.2). Fish samples were collected by diving and netting in coral reef habitats surrounding various islands of the Nansha Islands. Captured fish were transferred to the laboratory and stored at −20°C. Surface water samples were collected at each station using a water sampler, pre‐filtered through a 160 μm mesh, and subsequently filtered onto 0.7 μm glass fiber filters (GF/F) to collect phytoplankton.

### 2.2 Sampling

Fish samples were dissected within 2 months, during which their gut contents were meticulously collected for dietary analysis (Table 1). The gut contents of partial fish samples were identified at two magnifications (4 × 10 and 10 × 40) using a dissecting microscope. Due to the relatively small sample size and the high degree of digestion observed in many gut contents, it was not feasible to calculate composite dietary indices such as the index of relative importance (IRI). Instead, we assessed diet composition using frequency of occurrence (%F), which represents the proportion of individuals in which a given prey item was detected (Table 2).

$$\%F=\left(\frac{N_{i}}{N}\right)\times 100$$

where is the number of fish in which prey item i was found, and *N* is the total number of fish examined. %*F* represents the proportion of individuals containing a given prey type and provides a straightforward and interpretable measure of diet composition. This metric is widely considered to be a robust and interpretable measure for dietary analysis, particularly when sample sizes are limited or prey items are difficult to quantify (Baker et al. 2014).

**TABLE 1:** Summary of sample size, body size, and weight of the four carnivorous fish species.

Species	Sample size	Mean length (mm) ± SD	Range of length (mm)	mean Weight (g) ± SD	Range of weight (g)
*Gnathodentex aureolineatus*	19	13.4 ± 1.4	11.4 ~ 15.5	72.96 ± 19.47	41.84 ~ 103.22
*Lutjanus kasmira*	12	13.6 ± 1.7	12.0 ~ 17.9	81.13 ± 23.78	56.08 ~ 130.00
*Cephalopholis urodeta*	8	12.1 ± 1.4	10.0 ~ 14.2	59.85 ± 13.87	41.60 ~ 79.41
*Epinephelus hexagonatus*	13	12.0 ± 1.6	9.0 ~ 14.3	53.69 ± 19.73	20.76 ~ 91.28

**TABLE 2:** Frequency of occurrence (%F) of different prey categories in the gut contents of four fish species (frequency of occurrence [%F] refers to the proportion of individual fish in which a given prey item was detected).

Prey items	*G. aureolineatus*	*L. kasmira*	*C. urodeta*	*E. hexagonatus*
**Crustacean**				
Pistol shrimp	15.40%	0	0	0
Crab	46.20%	50.00%	33.30%	66.70%
Hermit crab	15.40%	0	0	0
**Mollusca**				
Gastropod	30.80%	0	0	0
Bivalve	15.40%	0	0	0
Bony fishes	15.40%	100.00%	33.30%	100.00%
Polychaetes	7.70%	0	0	0
Foraminifera	23.10%	0	0	0
Sea grass	0	50.00%	0	0

“Dorsal muscle tissue (1 g) was collected from each fish for stable isotope analysis.” The samples were freeze‐dried with lg‐1.0 vacuum freeze dryer (FLOM FD1600‐A) for 48 h, then the samples were ground with a mortar and pestle. The ground samples were placed in sample tubes, labeled, dried, and stored awaiting analysis on the instrument.

The gut contents were carefully extracted from the alimentary canal and the MP fecal DNA rapid extraction kit (FastDNA Spin kit for feces, MP biomedicals, Santa Ana, USA) was used to extract DNA from the contents of the digestive tract and dissolved in 30 μL Tris HCl (10 mmol·L^−1^, pH 8.0). The purity and content of DNA were detected by spectrophotometry (thermo nanodrop nd‐2000; Gene Company Limited, Waltham, MA, USA). The extracted DNA was stored at −20°C.

### 2.3 High‐Throughput Sequencing and Dietary OTU Analysis

A 313 bp fragment of the mitochondrial COI gene was amplified from each intestinal content sample using the universal PCR primer pair mlCOIintF/jgHCO2198 (Geller et al. 2013; Leray and Yang 2013). Leray et al. (2015) conducted experimental analyses on six different primer combinations and identified mlCOIintF/jgHCO2198 as the most effective, achieving an amplification success rate of 91%. Amplification at 20 μL system: 0.4 μL fastpfu polymerase, 2 μL 2.5 ^−1^mmol·L dNTPs, 4 μL5 × Fastpfu buffer, 5 μ 0.8 μmol·L^−1^ positive and negative primers respectively 50 μ. 10 ng DNA template. PCR amplification procedures were set as follows: pre‐denaturation at 95°C for 5 min, denaturation at 95°C for 30s, annealing at 55°C for 30s, and extension at 72°C for 45 s were carried out for 27 cycles. Finally, it was extended at 72°C for 10 min. After PCR products were detected by 2% agarose electrophoresis, HTS was performed on the illuminahiseq sequencing platform (Illumina, San Diego, CA, USA).

The raw data obtained from the Illumina MiSeq platform were subjected to quality control in accordance with the Illumina MiSeq/HiSeq platform workflow (Pompanon and Deagle 2012). After data splitting, removing primer sequences, and splicing paired‐end reads, the tags were filtered and intercepted. Only high‐quality, long (> 300 bp) sequences remained. To evaluate prey composition and diversity, the tags were clustered into operational taxonomic units (OTUs) with a 97% threshold. Those OTUs with a frequency of less than 10 were removed. Total richness was estimated by the OTU number, Shannon‐Wiener index, and Chao2 estimator to ensure that enough sequences were generated to cover the dietary spectrum of targets comprehensively.

Representative OTU sequences were aligned to GenBank sequences with Basic Local Alignment Search Tool (BLAST). The five top‐scoring BLAST hits were returned. If any of these were uncultured or unannotated sequences, then the next would be selected until a specific sequence series was compiled. A species name was accepted only if the similarity of the five best hits were ≥ 99%. A genus name was accepted only if the similarity of the five best hits were ≥ 98%. A family name was retained only if the similarities of all the best hits were ≥ 95%. Sequences with maximum similarities < 95% were labeled “NA (No Account).”. These were most likely the products of PCR errors, contamination, or GenBank deficiency. The number of effective tags was then returned for each phylum.

Additionally, Pianka's niche overlap index (Ojk) was applied to evaluate niche overlap among species (Soria‐Barreto and Rodiles‐Hernández 2008), based on relative abundances of prey OTUs. The analyses are presented as follows:

$$O_{\mathrm{jk}}=\frac{\sum_{i=1}^{n}p_{\mathrm{ij}}∙p_{\mathrm{ik}}}{\sqrt{\left(\sum_{i=1}^{n}p_{\mathrm{ij}}^{2}\right)∙\left(\sum_{i=1}^{n}p_{\mathrm{ik}}^{2}\right)}}$$

where *p*_*ij*_ and *p*_*ik*_ represent the proportional use of resource *i* by species *j* and *k*, respectively. The index ranges from 0 (no overlap) to 1 (complete overlap) and reflects the degree of dietary similarity between species (Vieira and Port 2007). Graphpad Prism 10 and Tutools (https://www.cloudtutu.com) were used for wayne diagram and mapping, and Adobe illustrator 2024 was used to edit maps.

### 2.4 Stable Isotope Analysis

**FIGURE 2:**
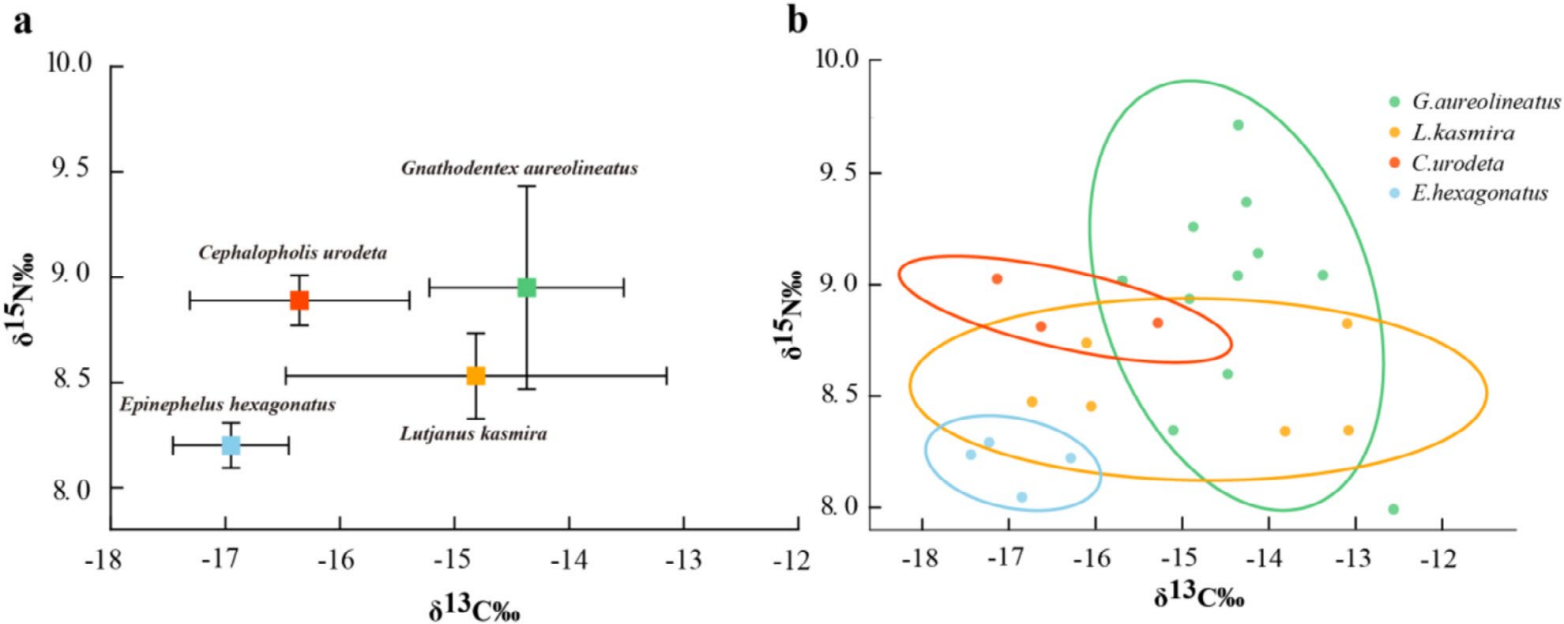
Stable isotope features of four fishes. (a) The isotopic distributions of δ^13^C and δ^15^N for four fish species (*Cephalopholis urodet*, *Epinephelus hexagonatus* , *Gnathodentex aureolineatus* , and *Lutjanus kasmira* ). Each point represents the mean value for a species, with error bars indicating the standard deviation. δ^13^C reflects the dietary carbon source, while δ^15^N indicates differences in trophic levels. (b) Isotope data are used to construct ellipses for the four fish species, with each ellipse representing the 95% confidence interval and distinguished by different colors. Each color corresponds to a specific fish species: Red squares (*C. urodet*), blue circles ( *E. hexagonatus* ), green triangles ( *G. aureolineatus* ), and yellow diamonds ( *L. kasmira* ).

δ^13^C and δ^15^N values were measured using an elemental analyzer (Flash2000; Thermo Fisher Scientific Inc., Italy) connected to an isotope ratio mass spectrometer (Delta V advantage; Thermo Fisher Scientific Inc., Germany) at the South China Sea Institute of Oceanography, Chinese Academy of Sciences, Guangzhou, China. The stable isotope ratio (‰) is calculated as δ^13^C or δ^15^N = [*R*_sample_/*R*_standard_ − 1] × 10^3^, where “δ” is ^13^C or ^15^N, and *R* is the ratio of ^13^C/^12^C or ^15^N/^14^N. The standard deviation in parts per thousands (‰) relative to the conventional C (Vienna Pee Dee Belemnite) and N (atmospheric N_2_) standard reference materials, and “R” is heavier to lighter isotopic ratios (^13^C/^12^C or ^15^N/^14^N) indicating ^15^C‐ and ^13^N‐enriched or depleted items. Protein (Casein) Standard (CatNo. B2155; Elemental Microanalysis Ltd., UK) was used as a certified reference material with an analytical precision of ±0.13‰ and ±0.08‰ for δ^13^C and δ^15^N values, respectively. A bi‐plot was conducted based on the δ^13^C and δ^15^N values of fish muscles (Figure 2a). Trophic levels of the fish species were calculated based on stable isotope data using the following formula:

$$\text{Trophic Level}=1+\frac{\delta^{15}N_{\text{consumer}}-\delta^{15}N_{\text{base}}}{∆\delta^{15}N}$$

where: *δ* ^*15*^ *N*_consumer_ is the nitrogen isotope value of the consumer (in this case, the fish species), *δ* ^*15*^ *N*_base_ is the nitrogen isotope value of the baseline producer (typically primary producers such as algae or primary consumers), *∆δ* ^*15*^ *N* is the typical nitrogen isotope enrichment factor (commonly assumed to be 3.4‰ (Wyatt et al. 2012), representing the enrichment per trophic level). The baseline producers in this study are represented by the primary producers, specifically phytoplankton from the study area. Stable isotope niche metrics were calculated using the Stable Isotope Bayesian Ellipses in R (SIBER) package (Layman 2007; Jackson et al. 2011) implemented in R (version 4.4.3). Standard ellipse areas corrected for small sample size (SEAc) were computed for each species using the SIBER function. SEAc represents the core isotopic niche area encompassing approximately 40% of the data and is less sensitive to outliers than convex hulls (Newsome et al. 2007). Total niche area (TA), defined as the area of the convex hull enclosing all data points for each group, was calculated using the SIBER package. To assess isotopic niche overlap between species, pairwise comparisons of standard ellipses were performed using polygon intersection methods. The proportion of overlap was calculated as the percentage of one group's ellipse area shared with another.

## 3 Results

### 3.1 Food Items Revealed by Dissecting Microscope Examination

The detection rate of crustaceans in the gut contents of the four fish species ranges from 33% to 77% (Table 2). *G. aureolineatus* had the highest recognizing food groups, including pistol shrimp, crab, hermit crab, gastropod, bivalve, fish, foraminifera, and annelida. Crabs also were found in other fish species, and the most common fragments are crab claws and legs. Fish bones were found in the gut contents of four fish, which means they all eat fish. Due to the high digestibility of gut contents of carnivorous fish, it is difficult to distinguish the accurate species of food by microscopic examination. The food photographs obtained can be referred to in Table S1.

### 3.2 Trophic Similarity and Dietary Overlap Among Four Species Revealed by Stable Isotope Analysis

Isotopic composition analysis was performed on 24 individuals of four fishes (Table 3). Among the four species, the highest δ^13^C value was *G. aureolineatus* (−14.4‰), and the lowest value was *E. hexagonatus* (−17.0‰). In addition, the maximum and minimum values of the mean values of δ^15^N were still *G. aureolineatus* (9.0‰) and *E. hexagonatus* (8.2‰).

**TABLE 3:** Stable isotope metrics and trophic levels of four carnivorous reef fish species.

Species	Sample size	Mean δ^13^C (‰ ± SE)	Mean δ^15^N (‰ ± SE)	SEAc((‰)^2^)	TA((‰)^2^)	Trophic level	Sampling area
*G. aureolineatus*	11	−14.37 ± 0.85	8.95 ± 0.48	1.351	2.76	3.6	NX, DM, CG, and HY
*L. kasmira*	6	−14.81 ± 1.66	8.53 ± 0.20	1.345	1.31	3.5	CG and YS
*C. urodeta*	3	−16.35 ± 0.96	8.89 ± 0.12	0.557	0.15	3.6	HY
*E. hexagonatus*	4	−16.95 ± 0.51	8.20 ± 0.11	0.237	0.13	3.4	ZB and NX

Substantial variation was observed in isotopic niche width, as measured by both SEAc and TA. *G. aureolineatus* exhibited the widest isotopic niche (SEAc = 1.351(‰)^2^, TA = 2.76(‰)^2^), indicating a broad range of dietary sources and high individual variation. *L. kasmira* displayed similarly high SEAc (1.345(‰)^2^) but a smaller TA (1.31(‰)^2^), suggesting a broad core diet with fewer extreme foraging outliers. Conversely, *C. urodeta* and *E. hexagonatus* exhibited considerably narrower isotopic niches (SEAc = 0.557(‰)^2^ and 0.237(‰)^2^, TA = 0.15(‰)^2^ and 0.13(‰)^2^, respectively), indicating more specialized foraging strategies and lower intrapopulation dietary variability. Niche overlap analysis based on SEAc ellipses indicated that significant isotopic niche overlap occurred only between *G. aureolineatus* and *L. kasmira* , with an overlapping area of 0.243(‰)^2^, corresponding to 20.0% and 22.6% of their respective niche areas (Figure 2b). All other species pairs showed no detectable ellipse overlap, indicating clear trophic segregation and minimal interspecific dietary redundancy.

Based on nitrogen stable isotope ratios and the trophic level calculation formula, the trophic levels of the four small carnivorous fish species in the Nansha Islands ranged from 3.4 to 3.6 (Table 3), with a maximum difference of only 0.2. *G. aureolineatus* and *C. urodeta* exhibited the highest trophic levels (3.6), followed by *L. kasmira* (3.5), while *E. hexagonatus* had the lowest (3.4).

### 3.3 High‐Throughput Sequencing Analysis

The sequencing results of the four fishes showed that 303,202 valid sequences were obtained, and 94,039 valid food sequences remained after removing the fishes' own sequences and unidentified sequences (Table 4). According to the comparison results, 47.37% of OTU identified species, 39.47% identified genera, and 13.16% of identified families and above.

**TABLE 4:** Summary of sequencing results and taxonomic identification of prey items based on gut content metabarcoding for four carnivorous fish species.

Samples	Total number of sequences	Number of effective sequences	Number of prey phylum	Number of prey species
*G. aureolineatus*	64,653	33,827	3	12
*L. kasmira*	80,937	2354	2	5
*C. urodeta*	53,883	51,988	2	8
*E. hexagonatus*	88,398	6872	2	5

38 OTUs were obtained after removing the fishes' own sequences, unidentifiable sequences and fewer sequences. These OTUs were divided into three phyla: Arthropoda, Mollusca, and Chordata. Arthropoda was the most abundant group among the four fishes, accounting for 85.7% of the total food sequence and containing 30 OTUs. Chordates accounted for 13.3% of the total food sequence and contained 7 OTUs. Mollusca contains only one OTU, accounting for 0.97%.

The food sequence of *G. aureolineatus* is at the phylum level: the phylum arthropod accounted for the vast majority (90.66%) of the total number of identifiable food sequences, among which the main species were the shrimps and crabs of the order Decapoda, such as *Penaeus* sp., *Alpheus dolerus* , *Thalamita seurati*, etc. A few sequences (5.86%) were *Undinula vulgaris* , a copepod. Fish and snails accounted for 6.66% and 2.68% of the food sequences.

The primary dietary components of *L. kasmira* in the field included *Calcinus latens* (41.55%), *Liocarpilodes pacificus* (41.12%), and *Etisus odhneri* (7.77%), along with members of the family Hippolytidae (41.55%) and *Lutjanus gibbus* (8.03%).

Seven food species were detected in the gut contents of *C. urodeta* , including Arthropods and Chordates, which were *Hippolytidae* sp. (30.96%), *Portunidae* sp. (19.06%), *Anomura* sp. (15.86%), and *Chromis xanthochira* (17.83%), etc.

The *E. hexagonatus* feeds on four types of food, which include *Hippolytidae* sp. (82.99%), *Neaxius glyptocercus* (2.78%), parrotfish *Scarus* sp. (11.34%), and *Pterocaesio tile* (2.9%), respectively.

**FIGURE 3:**
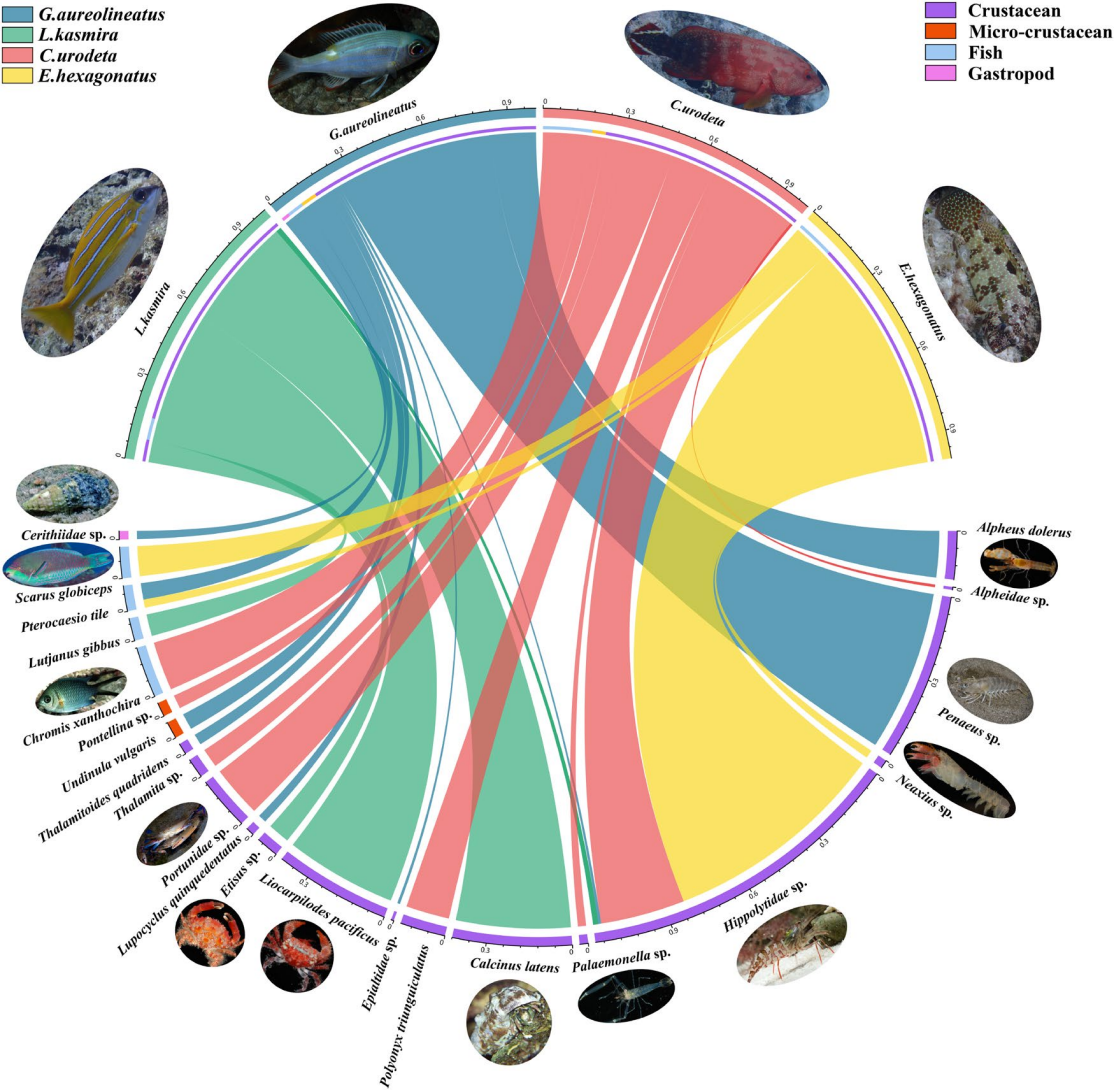
Dietary partitioning and composition of four fishes. The chord diagram illustrates the relationship between different fish species ( *Gnathodentex aureolineatus* , *Lutjanus kasmira* , *Cephalopholis urodet*, and *Epinephelus hexagonatus* ) and their main dietary components. Each fish species and its dietary categories are connected by colored chord lines, visually representing the feeding preferences and trophic interactions within the ecosystem. Fish Species (labeled segments at the top of the circle, differentiated by color): Green: *G. aureolineatus* , Yellow: *L. kasmira* , Red: *C. urodeta* , Blue: *E. hexagonatus*. Dietary Categories (labeled segments at the bottom of the circle, differentiated by color): Purple: Crustaceans, Pink: Microcrustaceans, Yellow: Bony fish, and Light Blue: Gastropods. Each chord represents the feeding relationship between a fish species and its dietary components, with the width of the chord reflecting the proportion of the diet. Specific prey species are shown alongside representative images to provide further clarity. The density and distribution of the chords visually highlight the primary dietary sources and trophic niche differentiation among the fish species.

The food sequences of the four fish species at the phylum level are as follows: Arthropods accounted for the vast majority (85.73%) of the total number of identifiable food sequences. In addition, fish accounted for 13.31% and snails accounted for 0.96% (Figure 3). Additionally, the majority of the dietary components (86.69%) are likely derived from benthos.

**FIGURE 4:**
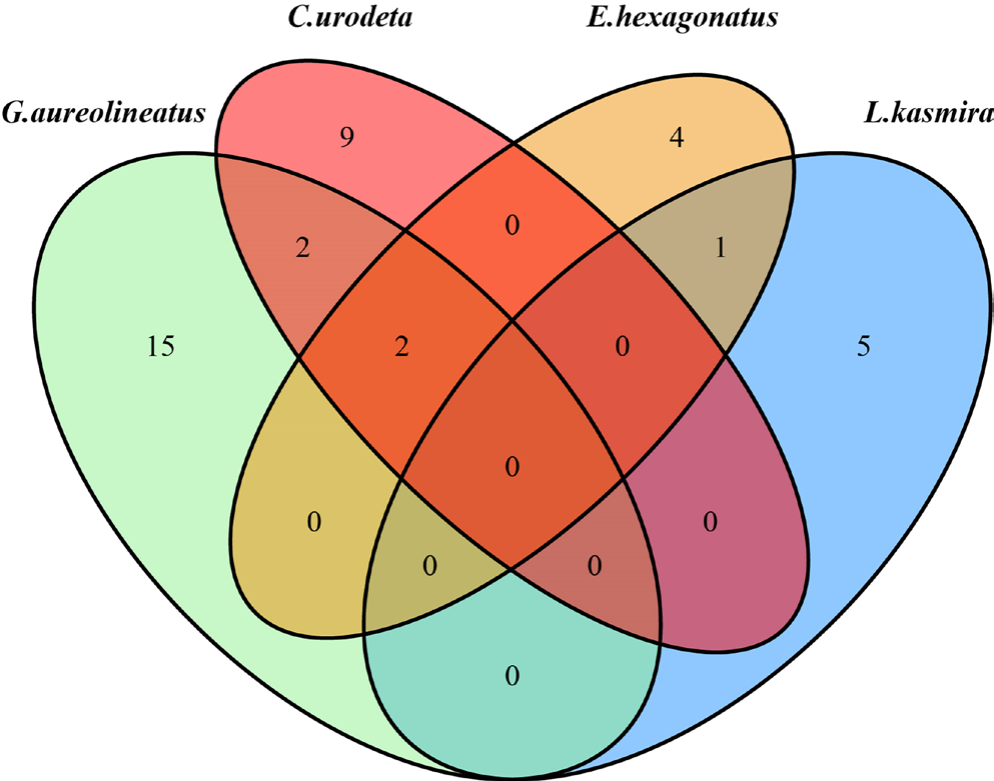
Venn diagram illustrating the overlap of dietary items (based on OTU counts) among four fish species ( *G. aureolineatus* , *L. kasmira* , *C. urodeta* , and *E. hegonatus*). The numbers in each section represent the number of unique or shared operational taxonomic units (OTUs) detected in the gut contents of each species. Non‐overlapping areas indicate prey OTUs specific to a single fish species, while overlapping areas indicate OTUs shared by two or more species.

The results show that there are 15 unique OTUs for *G. aureolineatus* , 9 unique OTUs for *C. urodeta* , 5 unique OTUs for *L. kasmira* , 4 unique OTUs for *E. hegonatus*, 2 common OTUs for *G. aureolineatus* and *C. urodeta* , 2 common OTUs for *G. aureolineatus* , *C. urodeta* , and *E. hegonatus*, and no overlapping OTUs for *L. kasmira* , and 1 common OTU for *L. kasmira* and *E. hegonatus* (Figure 4).

To assess dietary resource partitioning among the four reef‐associated fish species, pairwise Pianka's niche overlap indices were calculated based on OTU composition (Table 5). The results showed uniformly low overlap values, suggesting clear trophic segregation among species. All off‐diagonal values were well below 0.1, indicating that the fish species occupied distinct dietary niches with minimal resource redundancy, supporting the existence of strong trophic niche partitioning.

**TABLE 5:** Pairwise Pianka's niche overlap index among four reef‐associated fish species.

Pianka's niche overlap index	*G. aureolineatus*	*L. kasmira*	*C. urodeta*	*E. hexagonatus*
*G. aureolineatus*	1	0	0.009	0.001
*L. kasmira*	0	1	0	0.025
*C. urodeta*	0.009	0	1	0.046
*E. hexagonatus*	0.001	0.025	0.046	1

## 4 Discussion

This study provides new insights into the feeding ecology of carnivorous fish species inhabiting coral reef ecosystems, with a focus on their dietary preferences and trophic interactions. Our findings reveal that crustaceans, particularly crabs and shrimps, dominate the diet of the four fish species studied, with notable differences in prey species among them. Stable isotope analysis further validated the trophic positions of these species, revealing that they share similar yet distinct trophic niches within the reef ecosystem. The use of HTS technology enabled the identification of specific prey species, which highlighted the significant role of benthic organisms in supporting fish biomass.

### 4.1 Complementary Approaches to Trophic Assessment

Traditional gut content analysis has long been employed to investigate fish feeding behavior, providing direct evidence of ingested prey types. For example, Ali et al. (2016) reported that *L. kasmira* primarily consumed clupeid fish (frequency of occurrence: 89.34%) and secondarily crustaceans (10.66%) in the Socotrian Archipelago. Similarly, Nakai et al. (2001) found that *C. urodeta* predominantly fed on fish, followed by shrimp and crabs. However, the limitations of this method—particularly its inability to identify highly digested or morphologically indistinct prey—have been well documented. Zgliczynski et al. (2019) demonstrated this in their study of *Plectropomus oligacanthus* , where stable isotope analysis was required to complement gut content observations and revealed broader dietary components, including stomatopods and other invertebrates, which were difficult to detect microscopically. The apparent similarity in dietary profiles across islands was likely an artifact of the low taxonomic resolution afforded by traditional methods.

In our study, microscopic observations revealed that crustacean fragments—particularly crab appendages—were predominant in the gut contents of *G. aureolineatus* , *L. kasmira* , and *C. urodeta* , whereas such fragments were less frequently observed in *E. hexagonatus* . All four species contained fish scales, bones, and muscle tissue in their gut contents, suggesting a piscivorous diet. However, detailed taxonomic identification was hindered by digestion‐related degradation, reaffirming the methodological limitations noted in the literature.

To overcome these constraints, we applied HTS, which greatly improved taxonomic resolution and allowed for the identification of specific prey species. For example, we detected distinct crab taxa such as *Thalamitoides quadridens* , *Lupocyclus quinquedentatus* , and *Polyonyx triunguiculatus*, as well as prey fish including *P. tile* , *L. gibbus* , and *Chromis xanthochira* . These findings highlight the dietary breadth and interspecific variation among the four predators, which were not apparent using morphological analysis alone.

Our results are consistent with the growing body of literature supporting multi‐method approaches in trophic ecology. Wyatt et al. (2012) showed that stable isotopes and fatty acid analyses can differentiate between oceanic and reef‐based productivity sources across fish trophic guilds. Similarly, Yin et al. (2023) revealed significant seasonal shifts in coral reef food webs using stable isotope analysis, emphasizing the importance of benthic macroalgae and microalgae as basal resources. Furthermore, recent studies have effectively combined gut content analysis, stable isotopes, and DNA metabarcoding to detect fine‐scale dietary variation and niche differentiation in reef fishes (Wu et al. 2022; Lin et al. 2023).

By integrating HTS with traditional gut content examination, our study not only confirmed crustaceans as the dominant prey group but also revealed fine‐scale taxonomic distinctions and niche divergence among co‐occurring carnivorous fish species. This methodological synergy enhances our capacity to assess species‐specific trophic roles and provides a more comprehensive understanding of predator–prey interactions within coral reef ecosystems.

### 4.2 The Differences in Food Resource Utilization of Four Fishes

Our results revealed that all four carnivorous fish species primarily consumed crustaceans, though with notable interspecific differences in prey composition. *G. aureolineatus* and *C. urodeta* predominantly fed on penaeid shrimp and portunid crabs, while *L. kasmira* and *E. hexagonatus* showed a higher proportion of xanthid and hippolytid species, respectively.

Previous studies have similarly reported crustaceans as key dietary components for *L. kasmira* and *C. urodeta* , although fish prey often dominate by weight (Nakai et al. 2001; Ali et al. 2016). In contrast, our gut content analysis revealed a higher frequency of crustacean remains, particularly crab appendages, suggesting a stronger crustacean preference than previously reported. This discrepancy may be attributed to regional prey availability or size‐dependent dietary shifts.

Our findings are further supported by HTS, which enabled species‐level resolution of crustacean prey—such as *C. latens* , *T. quadridens* , and *P. triunguiculatus*—that were previously indistinguishable through morphological analysis. The repeated presence of algal‐associated shrimp (e.g., Hippolytidae, *Palaemonella* sp.) across species highlights the ecological importance of small cryptic crustaceans as shared prey resources in coral reef systems. These taxa often co‐occur with macroalgae or corals and contribute significantly to reef trophic connectivity (Xu 2014; Rouzé et al. 2014; Palomares and Pauly 2024).

Trophic level estimates based on δ^15^N values ranged narrowly from 3.4 to 3.6. However, substantial differences were observed in isotopic niche width among species, as indicated by SEAc and TA metrics. *G. aureolineatus* exhibited the broadest isotopic niche (SEAc = 1.351(‰)^2^, TA = 2.76(‰)^2^), suggesting a diverse array of dietary sources and high individual‐level dietary variation. *L. kasmira* showed a similarly wide SEAc (1.345(‰)^2^) but a smaller TA (1.31(‰)^2^), indicating a relatively broad core diet with fewer individuals exhibiting extreme foraging behavior. These results align with the view that isotopic niche width reflects long‐term dietary variation, even when short‐term prey observations suggest narrower specialization (Newsome et al. 2007). Differences in prey trophic positions—such as the more herbivorous diet of xanthid crabs versus the carnivorous habits of portunids—may also help explain the observed patterns (Choy 1986; Safaie 2016).

In contrast, *C. urodeta* and *E. hexagonatus* displayed much narrower isotopic niches (SEAc = 0.557(‰)^2^ and 0.237(‰)^2^; TA = 0.15(‰)^2^ and 0.13(‰)^2^, respectively), implying more specialized foraging strategies and lower intraspecific variability in diet. These patterns are consistent with gut content and HTS data, which together revealed both shared prey preferences and species‐specific dietary tendencies. HTS further enabled species‐level resolution of crustacean prey—such as *C. latens* , *T. quadridens* , and *P. triunguiculatus*—that were previously indistinguishable through morphological analysis. The repeated presence of algal‐associated shrimp (e.g., Hippolytidae, *Palaemonella* sp.) across species highlights the ecological importance of small cryptic crustaceans as shared prey resources in coral reef systems. These taxa often co‐occur with macroalgae or corals and contribute significantly to reef trophic connectivity (Xu 2014; Rouzé et al. 2014; Palomares and Pauly 2024). Niche overlap analysis based on SEAc ellipses revealed significant overlap only between *G. aureolineatus* and *L. kasmira* , suggesting partial dietary convergence between these two species. In contrast, the absence of isotopic ellipse overlap among other species pairs points to clear trophic segregation and minimal interspecific dietary redundancy.

It is also important to acknowledge a key limitation of this study: the fish samples were collected from multiple reef systems across the Nansha Islands. These reef environments likely differ in oceanographic and ecological conditions, which may lead to spatial variation in isotopic baselines—particularly in δ^13^C values. Such variation could influence not only absolute isotopic values but also the derived estimates of isotopic niche width (SEAc and TA). Therefore, some interspecific differences in isotopic niche size may partially reflect underlying environmental differences rather than purely dietary specialization. Although we were unable to apply baseline corrections due to the constraints of the sampling design, the integration of gut content morphology, stable isotope analysis, and DNA metabarcoding provides a robust, multidimensional framework for evaluating trophic interactions and resource partitioning among reef mesopredators in a spatially heterogeneous ecosystem.

### 4.3 Dietary Shifts and Responses to Coral Reef Change

In addition to crustaceans, all four carnivorous fish species were found to consume teleosts, although with differing prey identities. *G. aureolineatus* and *E. hexagonatus* fed on *P. tile* , while *L. kasmira* and *C. urodeta* preyed on *L. gibbus* and *C. xanthochira* , respectively. Given the size disparity between predators and prey, these fish were likely consuming larval or juvenile stages. This aligns with earlier findings that *L. kasmira* preferentially targets fish larvae (Ali et al. 2016; Mablouké et al. 2013) and tends to forage in the water column (Rangarajan 1970), though our results indicate a shift toward crustacean dominance, possibly driven by local prey availability.

Interestingly, while FishBase and prior studies have emphasized clupeid dominance in the diet of *L. kasmira* (e.g., *Encrasicholina punctifer* ; Ali et al. 2016), our surveys detected no clupeid or engraulid species in the Nansha reef region. This absence may signal regional declines in small pelagic fish, potentially due to overfishing or habitat degradation, as suggested by decreasing feeding intensities and body conditions in *L. kasmira* and *G. aureolineatus* from 2016 to 2019 (Zhang et al. 2023).

Despite the observed isotopic niche overlap between *G. aureolineatus* and *L. kasmira* , DNA‐based diet profiling revealed substantial differentiation at the prey species level. All species relied on crustaceans, but the taxonomic composition varied: penaeids and portunids dominated the diet of *G. aureolineatus* , whereas *L. kasmira* fed primarily on xanthids and hermit crabs. This fine‐scale dietary divergence likely contributes to reducing interspecific competition within the shared reef habitat, consistent with niche partitioning theory in tropical fish communities (Schoener 1983; Ross 1986).

Coral reef degradation may intensify food competition by reducing structural complexity and the availability of benthic prey (Taira et al. 2020). However, recent studies have shown that turf‐covered dead coral can support dense populations of harpacticoid copepods and small invertebrates (Fraser et al. 2021; Kramer et al. 2014). Our findings suggest that carnivorous fishes may flexibly adjust prey composition in response to benthic shifts, with species‐specific preferences for either decapods or copepods. Such trophic plasticity, together with subtle dietary segregation, may promote the coexistence of mesopredators under dynamic reef conditions.

## 5 Conclusion

This study reveals that crustaceans, rather than fish, constitute the predominant dietary component of four mesopredatory reef fishes ( *G. aureolineatus* , *L. kasmira* , *C. urodeta* , and *E. hexagonatus* ) in the degraded coral reef ecosystems of the Nansha Islands. By integrating gut content morphology, HTS, and stable isotope analysis, we demonstrate species‐specific prey preferences and fine‐scale dietary partitioning, despite apparent overlaps in isotopic niches. These findings suggest that benthic crustaceans not only represent a major trophic resource but also facilitate the coexistence of sympatric predators through niche differentiation. Moreover, the complementary application of multiple analytical approaches enhances our capacity to resolve trophic relationships that may be masked by individual methods alone. Under ongoing coral reef degradation, small benthic invertebrates—particularly decapods and copepods—may play a disproportionately important role in maintaining predator populations and ecosystem stability.

## Author Contributions

**Chen Zhang:** conceptualization (equal), data curation (equal), formal analysis (equal), software (equal), visualization (lead), writing – original draft (lead), writing – review and editing (equal). **Simin Hu:** conceptualization (equal), data curation (equal), methodology (equal), project administration (equal), writing – review and editing (equal). **Xianzhi Lin:** conceptualization (equal), data curation (equal), investigation (equal), methodology (equal), writing – review and editing (equal). **Hui Huang:** conceptualization (equal), data curation (equal), project administration (equal), resources (equal), writing – review and editing (equal). **Sheng Liu:** conceptualization (equal), data curation (equal), funding acquisition (equal), investigation (equal), project administration (equal), resources (equal), writing – review and editing (equal).

## Ethics Statement

The fish study was reviewed and approved by the Laboratory Animal Management and Ethics Committee, South China Sea Institute of Oceanology, Chinese Academy of Sciences (ID:LMB2024110601).

## Conflicts of Interest

The authors declare no conflicts of interest.

## Supporting information


**Table S1.** Food items observed by microscopic in the gut contents of fishes from the Nansha coral reefs.

## Data Availability

The data associated with this article has been deposited in the Science data bank and are available at https://www.scidb.cn/en/anonymous/Sk5KZnV5.

## References

[ece371497-bib-0001] Ali, M. , A. Belluscio , D. Ventura , and G. Ardizzone . 2016. “Feeding Ecology of Some Fish Species Occurring in Artisanal Fishery of Socotra Island (Yemen).” Marine Pollution Bulletin 105, no. 2: 613–628. 26880127 10.1016/j.marpolbul.2016.01.051

[ece371497-bib-0002] Almany, G. R. 2004. “Does Increased Habitat Complexity Reduce Predation and Competition in Coral Reef Fish Assemblages?” Oikos 106, no. 2: 275–284.

[ece371497-bib-0003] Baker, R. , A. Buckland , and M. Sheaves . 2014. “Fish Gut Content Analysis: Robust Measures of Diet Composition.” Fish and Fisheries 15, no. 1: 170–177.

[ece371497-bib-0006] Brandl, S. J. , J. M. Casey , and C. P. Meyer . 2020. “Dietary and Habitat Niche Partitioning in Congeneric Cryptobenthic Reef Fish Species.” Coral Reefs 39, no. 2: 305–317.

[ece371497-bib-0008] Choat, J. H. , K. D. Clements , and A. W. Robbins . 2002. “The Trophic Status of Herbivorous Fishes on Coral Reefs—I: Dietary Analyses.” Marine Biology 140, no. 3: 613–623.

[ece371497-bib-0009] Choy, S. C. 1986. “Natural Diet and Feeding Habits of the Crabs *Liocarcinus puber* and *L. holsatus* (Decapoda, Brachyura, Portunidae).” Marine Ecology Progress Series 31, no. 6: 87–99.

[ece371497-bib-0010] Cook, K. M. , H. Yamagiwa , M. Beger , et al. 2022. “A Community and Functional Comparison of Coral and Reef Fish Assemblages Between Four Decades of Coastal Urbanisation and Thermal Stress.” Ecology and Evolution 12, no. 3: e8736. 35356574 10.1002/ece3.8736 PMC8939291

[ece371497-bib-0012] Darling, E. S. , N. A. Graham , F. A. Januchowski‐Hartley , K. L. Nash , M. S. Pratchett , and S. K. Wilson . 2017. “Relationships Between Structural Complexity, Coral Traits, and Reef Fish Assemblages.” Coral Reefs 36, no. 2: 561–575.

[ece371497-bib-0013] Deagle, B. E. , A. C. Thomas , J. C. McInnes , et al. 2019. “Counting With DNA in Metabarcoding Studies: How Should We Convert Sequence Reads to Dietary Data?” Molecular Ecology 28, no. 2: 391–406. 29858539 10.1111/mec.14734 PMC6905394

[ece371497-bib-0014] Deagle, B. E. , A. C. Thomas , A. K. Shaffer , A. W. Trites , and S. N. Jarman . 2013. “Quantifying Sequence Proportions in a DNA‐Based Diet Study Using Ion Torrent Amplicon Sequencing: Which Counts Count?” Molecular Ecology Resources 13, no. 4: 620–633. 23590207 10.1111/1755-0998.12103

[ece371497-bib-0016] Dias, R. M. , R. M. Tófoli , J. C. B. da Silva , L. C. Gomes , and A. A. Agostinho . 2022. “Effects of Habitat Complexity on Trophic Interactions of Three Congeneric Fish Species.” Aquatic Ecology 56, no. 3: 877–889.

[ece371497-bib-0017] Enochs, I. C. , and D. P. Manzello . 2012. “Species Richness of Motile Cryptofauna Across a Gradient of Reef Framework Erosion.” Coral Reefs 31, no. 3: 653–661.

[ece371497-bib-0018] Fraser, K. M. , R. D. Stuart‐Smith , S. D. Ling , and G. J. Edgar . 2021. “High Biomass and Productivity of Epifaunal Invertebrates Living Amongst Dead Coral.” Marine Biology 168, no. 7: 102.

[ece371497-bib-0019] Froese, R. , and D. Pauly . 2024. “FishBase, World Wide Web Electronic Publication.”

[ece371497-bib-0020] Geller, J. , C. Meyer , M. Parker , and H. Hawk . 2013. “Redesign of PCR Primers for Mitochondrial Cytochrome *c* Oxidase Subunit I for Marine Invertebrates and Application in All‐Taxa Biotic Surveys.” Molecular Ecology Resources 13, no. 5: 851–861. 23848937 10.1111/1755-0998.12138

[ece371497-bib-0021] Graham, N. A. J. , and K. L. Nash . 2013. “The Importance of Structural Complexity in Coral Reef Ecosystems.” Coral Reefs 32, no. 2: 315–326.

[ece371497-bib-0022] Head, C. , M. B. Bonsall , C. E. I. Head , et al. 2018. “Exceptional Biodiversity of the Cryptofaunal Decapods in the Chagos Archipelago, Central Indian Ocean.” Marine Pollution Bulletin 135: 636–647. 30301083 10.1016/j.marpolbul.2018.07.063

[ece371497-bib-0023] Hiatt, R. W. , and D. W. Strasburg . 1960. “Ecological Relationships of the Fish Fauna on Coral Reefs of the Marshall Islands.” Ecological Monographs 30, no. 1: 65–127.

[ece371497-bib-0024] Hixon, M. A. , and J. P. Beets . 1993. “Predation, Prey Refuges, and the Structure of Coral‐Reef Fish Assemblages.” Ecological Monographs 63, no. 1: 77–101.

[ece371497-bib-0025] Hu, S. M. , Z. L. Guo , S. Hu , et al. 2015. “Molecular Analysis of *In Situ* Diets of Coral Reef Copepods: Evidence of Terrestrial Plant Detritus as a Food Source in Sanya Bay, China.” Journal of Plankton Research 37, no. 2: 363–371.

[ece371497-bib-0026] Huanting, Z. , and W. Lirong . 2017. “Natural Environment, Resources and Development of the South China Sea Islands: The 70th Anniversary of Recovery of the South China Sea Islands.” Tropical Geography 37, no. 5: 659–680.

[ece371497-bib-0027] Jackson, A. L. , R. Inger , A. C. Parnell , and S. Bearhop . 2011. “Comparing Isotopic Niche Widths Among and Within Communities: SIBER—Stable Isotope Bayesian Ellipses in R.” Journal of Animal Ecology 80, no. 3: 595–602. 21401589 10.1111/j.1365-2656.2011.01806.x

[ece371497-bib-0028] Kochan, D. P. , M. D. Mitchell , R. Zuercher , and A. R. Harborne . 2023. “Winners and Losers of Reef Flattening: An Assessment of Coral Reef Fish Species and Traits.” Oikos 2023, no. 12: e10011.

[ece371497-bib-0029] Komyakova, V. , P. L. Munday , and G. P. Jones . 2013. “Relative Importance of Coral Cover, Habitat Complexity and Diversity in Determining the Structure of Reef Fish Communities.” PLoS One 8, no. 12: e83178. 24349455 10.1371/journal.pone.0083178 PMC3862682

[ece371497-bib-0030] Kramer, M. J. , D. R. Bellwood , and O. Bellwood . 2014. “Benthic Crustacea on Coral Reefs: A Quantitative Survey.” Marine Ecology Progress Series 511: 105–116.

[ece371497-bib-0031] Kramer, M. J. , D. R. Bellwood , R. B. Taylor , and O. Bellwood . 2017. “Benthic Crustacea From Tropical and Temperate Reef Locations: Differences in Assemblages and Their Relationship With Habitat Structure.” Coral Reefs 36, no. 3: 971–980.

[ece371497-bib-0032] Kramer, M. J. , O. Bellwood , C. J. Fulton , and D. R. Bellwood . 2015. “Refining the Invertivore: Diversity and Specialisation in Fish Predation on Coral Reef Crustaceans.” Marine Biology 162, no. 9: 1779–1786.

[ece371497-bib-0034] Layman, C. A. 2007. “What Can Stable Isotope Ratios Reveal About Mangroves as Fish Habitat?” Bulletin of Marine Science 80, no. 3: 513–527.

[ece371497-bib-0036] Leray, M. , A. L. Alldredge , J. Y. Yang , et al. 2019. “Dietary Partitioning Promotes the Coexistence of Planktivorous Species on Coral Reefs.” Molecular Ecology 28, no. 10: 2694–2710. 30933383 10.1111/mec.15090 PMC6852152

[ece371497-bib-0037] Leray, M. , C. Meyer , C. P. Meyer , and S. C. Mills . 2015. “Metabarcoding Dietary Analysis of Coral Dwelling Predatory Fish Demonstrates the Minor Contribution of Coral Mutualists to Their Highly Partitioned, Generalist Diet.” PeerJ 3: e1047. 26137428 10.7717/peerj.1047 PMC4485734

[ece371497-bib-0038] Leray, M. , and J. Y. Yang . 2013. “A New Versatile Primer Set Targeting a Short Fragment of the Mitochondrial COI Region for Metabarcoding Metazoan Diversity: Application for Characterizing Coral Reef Fish Gut Contents.” Frontiers in Zoology 10: 1–14. 23767809 10.1186/1742-9994-10-34 PMC3686579

[ece371497-bib-0039] Li, Y. J. , and J. Zhang . 2020. “Study on Taxonomic Diversity of Fish in Zhubi Reef of Nansha Islands.” South China Fisheries Science 16, no. 1: 36–41.

[ece371497-bib-0041] Lin, X. , S. Hu , Y. Zhou , H. Huang , L. Zhang , and S. Liu . 2023. “A Multiple‐Methods Approach to Investigate Dietary Differences Among Nominally Herbivorous Fishes.” Marine Biology 170, no. 11: 134.

[ece371497-bib-0042] Lin, X. Z. , S. M. Hu , X. Lin , S. Hu , S. Liu , and H. Huang . 2018. “Unexpected Prey of Juvenile Spotted Scat (*Scatophagus argus*) Near a Wharf: The Prevalence of Fouling Organisms in Stomach Contents.” Ecology and Evolution 8, no. 16: 8547–8554. 30250722 10.1002/ece3.4380 PMC6145014

[ece371497-bib-0044] Mablouké, C. , J. Kolasinski , M. Potier , et al. 2013. “Feeding Habits and Food Partitioning Between Three Commercial Fish Associated With Artificial Reefs in a Tropical Coastal Environment.” African Journal of Marine Science 35, no. 3: 323–334.

[ece371497-bib-0045] McCutchan, J. H., Jr. , W. M. Lewis Jr. , C. Kendall , and C. C. McGrath . 2003. “Variation in Trophic Shift for Stable Isotope Ratios of Carbon, Nitrogen, and Sulfur.” Oikos 102, no. 2: 378–390.

[ece371497-bib-0047] Messmer, V. , G. P. Jones , P. L. Munday , S. J. Holbrook , R. J. Schmitt , and A. J. Brooks . 2011. “Habitat Biodiversity as a Determinant of Fish Community Structure on Coral Reefs.” Ecology 92, no. 12: 2285–2298. 22352168 10.1890/11-0037.1

[ece371497-bib-0048] Mills, M. S. , T. Schils , A. D. Olds , and J. X. Leon . 2023. “Structural Complexity of Coral Reefs in Guam, Mariana Islands.” Remote Sensing 15, no. 23: 5558.

[ece371497-bib-0049] Minagawa, M. , D. A. Winter , and I. R. Kaplan . 1984. “Comparison of Kjeldahl and Combustion Methods for Measurement of Nitrogen Isotope Ratios in Organic Matter.” Analytical Chemistry 56, no. 11: 1859–1861.

[ece371497-bib-0050] Mumby, P. J. , C. P. Dahlgren , A. R. Harborne , et al. 2006. “Fishing, Trophic Cascades, and the Process of Grazing on Coral Reefs.” Science 311, no. 5757: 98–101. 16400152 10.1126/science.1121129

[ece371497-bib-0051] Mumby, P. J. , R. S. Steneck , A. J. Edwards , et al. 2012. “Fishing Down a Caribbean Food Web Relaxes Trophic Cascades.” Marine Ecology Progress Series 445: 13–24.

[ece371497-bib-0052] Nakai, T. , M. Sano , and H. Kurokura . 2001. “Feeding Habits of the Darkfin Hind *Cephalopholis urodeta* (Serranidae) at Iriomote Island, Southern Japan.” Fisheries Science 67, no. 4: 640–643.

[ece371497-bib-0054] Newsome, S. D. , C. Martinez del Rio , S. Bearhop , and D. L. Phillips . 2007. “A Niche for Isotopic Ecology.” Frontiers in Ecology and the Environment 5, no. 8: 429–436.

[ece371497-bib-0055] Pace, M. L. 2013. “Trophic Cascades.” In Encyclopedia of Biodiversity, edited by S. A. Levin , Second ed., 258–263. Academic Press.

[ece371497-bib-0056] Palomares, M. L. D. , and D. Pauly . 2024. “SeaLifeBase, World Wide Web Electronic Publication.”

[ece371497-bib-0058] Pompanon, F. , and B. E. Deagle . 2012. “Who Is Eating What: Diet Assessment Using Next Generation Sequencing.” Molecular Ecology 21, no. 8: 1931–1950. 22171763 10.1111/j.1365-294X.2011.05403.x

[ece371497-bib-0059] Post, D. M. 2002. “Using Stable Isotopes to Estimate Trophic Position: Models, Methods, and Assumptions.” Ecology 83, no. 3: 703–718.

[ece371497-bib-0060] Rangarajan, K. 1970. “Food and Feeding Habits of the Snapper, *Lutianus kasmira* (Forskal) From the Andaman Sea.” Indian Journal of Fisheries 17: 43–52.

[ece371497-bib-0061] Roberts, C. M. , and R. Ormond . 1987. “Habitat Complexity and Coral‐Reef Fish Diversity and Abundance on Red‐Sea Fringing Reefs.” Marine Ecology Progress Series 41, no. 1: 1–8.

[ece371497-bib-0062] Ross, S. T. 1986. “Resource Partitioning in Fish Assemblages—a Review of Field Studies.” Copeia 2: 352–388.

[ece371497-bib-0063] Rouzé, H. , G. Lecellier , S. C. Mills , S. Planes , V. Berteaux‐Lecellier , and H. Stewart . 2014. “Juvenile *Trapezia* spp. Crabs Can Increase Juvenile Host Coral Survival by Protection From Predation.” Marine Ecology Progress Series 515: 151–159.

[ece371497-bib-0064] Safaie, M. 2016. “Feeding Habits of Blue Swimming Crab Portunus Segnis (Forskal, 1775) in the Northern Coastal Waters of Iran.” Marine Biodiversity Records 9: 1–9.

[ece371497-bib-0065] Schoener, T. W. 1983. “Field Experiments on Interspecific Competition.” American Naturalist 122, no. 2: 240–285.

[ece371497-bib-0066] Shao, K. T. 2024. “Taiwan Fish Database, WWW Web Electronic Publication.”

[ece371497-bib-0067] Soria‐Barreto, M. , and R. Rodiles‐Hernández . 2008. “Spatial Distribution of Cichlids in Tzendales River, Biosphere Reserve Montes Azules, Chiapas, Mexico.” Environmental Biology of Fishes 83: 459–469.

[ece371497-bib-0068] Stewart, B. D. , and G. P. Jones . 2001. “Associations Between the Abundance of Piscivorous Fishes and Their Prey on Coral Reefs: Implications for Prey‐Fish Mortality.” Marine Biology 138: 383–397.

[ece371497-bib-0069] Strona, G. , K. D. Lafferty , S. Fattorini , et al. 2021. “Global Tropical Reef Fish Richness Could Decline by Around Half if Corals Are Lost.” Proceedings of the Royal Society B: Biological Sciences 288: 20210274. 10.1098/rspb.2021.0274 PMC8242923 34187190

[ece371497-bib-0070] Taira, D. , E. C. Heery , L. H. L. Loke , A. Teo , A. G. Bauman , and P. A. Todd . 2020. “Ecological Engineering Across Organismal Scales: Trophic‐Mediated Positive Effects of Microhabitat Enhancement on Fishes.” Marine Ecology Progress Series 656: 181–192.

[ece371497-bib-0072] Tonn, W. M. , C. A. Paszkowski , and I. J. Holopainen . 1992. “Piscivory and Recruitment: Mechanisms Structuring Prey Populations in Small Lakes.” Ecology 73, no. 3: 951–958.

[ece371497-bib-0073] Vieira, E. M. , and D. Port . 2007. “Niche Overlap and Resource Partitioning Between Two Sympatric Fox Species in Southern Brazil.” Journal of Zoology 272, no. 1: 57–63.

[ece371497-bib-0074] Wang, L. , and S. Hu . 2020. “In Situ Feeding Differences Between Adults and Juveniles of Chaetognath (*Flaccisagitta enflata*) in Sanya Bay.” Journal of Tropical Oceanography 39, no. 3: 57–65.

[ece371497-bib-0075] Ward, A. J. , M. M. Webster , and P. J. Hart . 2006. “Intraspecific Food Competition in Fishes.” Fish and Fisheries 7, no. 4: 231–261.

[ece371497-bib-0076] Wilson, S. K. , S. C. Burgess , A. J. Cheal , et al. 2008. “Habitat Utilization by Coral Reef Fish: Implications for Specialists vs. Generalists in a Changing Environment.” Journal of Animal Ecology 4: 220–228. 10.1111/j.1365-2656.2007.01341.x 18081778

[ece371497-bib-0077] Wu, P. , T. Wang , Y. Liu , et al. 2022. “Differences of Macroalgal Consumption by Eight Herbivorous Coral Reef Fishes From the Xisha Islands, China.” Frontiers in Marine Science 9: 882196.

[ece371497-bib-0079] Wyatt, A. S. , A. M. Waite , and S. Humphries . 2012. “Stable Isotope Analysis Reveals Community‐Level Variation in Fish Trophodynamics Across a Fringing Coral Reef.” Coral Reefs 31, no. 4: 1029–1044.

[ece371497-bib-0080] Xu, P. 2014. “Study on the Taxonomy and Zoogeography of the Hippolytidae (Crustacea: Decapoda) of China Seas, The University of Chinese Academy of Sciences.”

[ece371497-bib-0082] Yin, H. , Y. Chen , W. Ma , et al. 2023. “δ^13^C and δ^15^N Stable Isotopes Demonstrate Seasonal Changes in the Food Web of Coral Reefs at the Wuzhizhou Island of the South China Sea.” Ecological Indicators 146: 109852.

[ece371497-bib-0083] Zanden, M. J. V. , and J. B. Rasmussen . 2001. “Variation in δ^15^N and δ^13^C Trophic Fractionation: Implications for Aquatic Food Web Studies.” Limnology and Oceanography 46, no. 8: 2061–2066.

[ece371497-bib-0084] Zgliczynski, B. J. , G. J. Williams , S. L. Hamilton , et al. 2019. “Foraging Consistency of Coral Reef Fishes Across Environmental Gradients in the Central Pacific.” Oecologia 191, no. 2: 433–445. 31485849 10.1007/s00442-019-04496-9

[ece371497-bib-0085] Zhang, C. , and S. Hu . 2022. “Diet and Trophic Level Analysis of Triggerfish (*Balistapus undulatuse*) in Coral Reefs of Nansha.” Journal of Tropical Oceanography 41, no. 1: 7–14.

[ece371497-bib-0086] Zhang, J. , Y. Cai , J. Li , et al. 2023. “Changes in Population Biology of Three Coral Reef Fishes in the South China Sea Between 1998–1999 and 2016–2019.” Frontiers in Conservation Science 4: 1129266.

[ece371497-bib-0087] Zhang, J. , and Z. Chen . 2015. “A Preliminary Study on Biology of Glowfish (*Gnathodentex aureolineatus*)in Yongshu Reef of Nansha Area in the South China Sea.” South China Fisheries Science 11, no. 5: 108–116.

