# Correction to “Crustaceans as Key Prey: Insights Into the Dietary Partitioning of Four Carnivorous Fishes in the Nansha Islands, South China Sea”

**DOI:** 10.1002/ece3.71690

**Published:** 2025-07-07

Zhang, C., Hu, S., Lin, X., Huang, H. and Liu, S. (2025), Crustaceans as Key Prey: Insights Into the Dietary Partitioning of Four Carnivorous Fishes in the Nansha Islands, South China Sea. *Ecology and Evolution*, 15: e71497. https://doi.org/10.1002/ece3.71497

The authors would like to correct the funding statement in the published article as it is incorrect.

**The original statement:**

“This work was supported by National Science and Technology Fundamental Resources Investigation Program of China (No. 2022FY100602), National Key Research and Development Program (No. 2021YFC3100500; 2022YFC3103605), Science and Technology Planning Project of Guangzhou, China (2023A04J0199), National Natural Science Foundation of China (No. 42176118), Special Fund of South China Sea Institute of Oceanology of the Chinese Academy of Sciences (No. SCSIO2023QY04), Science and Technology Planning Project of Guangdong Province, China (No. 2023B1212060047).”

**should be corrected to:**

“This work was supported by the National Key Research and Development Program (No. 2022YFC3103605); the National Natural Science Foundation of China (No. 42176118); the Special Fund of South China Sea Institute of Oceanology of the Chinese Academy of Sciences (No. SCSIO2023QY04); and the Science and Technology Planning Project of Guangdong Province, China (No. 2023B1212060047).”

We apologize for this error.

