# Supplementary material for: Crustaceans as Key Prey: Insights Into the Dietary Partitioning of Four Carnivorous Fishes in the Nansha Islands, South China Sea
Source: Ecol Evol. 2025 Jun 4;15(6):e71497. doi: 10.1002/ece3.71497 (PMC12137187; doi:10.1002/ece3.71497)
Supplement: Supplementary file 1 — Table S1. Food items observed by microscopic in the gut contents of fishes from the Nansha coral reefs. [file ECE3-15-e71497-s001.docx]

**Support information**

**Table S1 Food items observed by microscopic in the gut contents of fishes from the Nansha coral reefs.**

| Fish species | Food groups observed | Photographs of food items in fish gut contents |
| --- | --- | --- |
| *G.aureolineatus* | Pistol Shrimp, Crabs, Hermit Crabs, Gastropods, Bivalve, Fish, Foraminifera, Annelida | 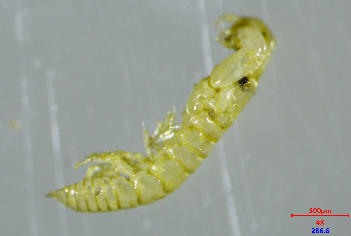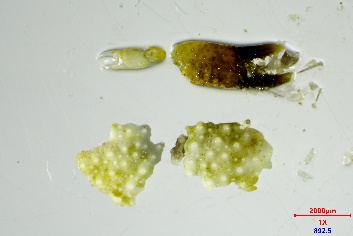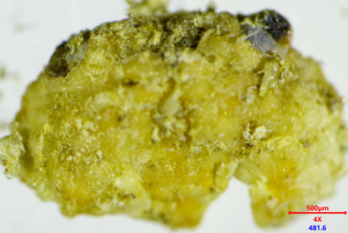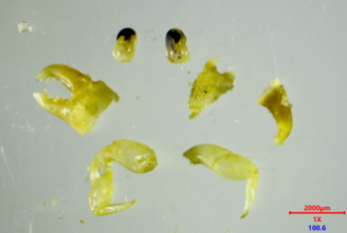  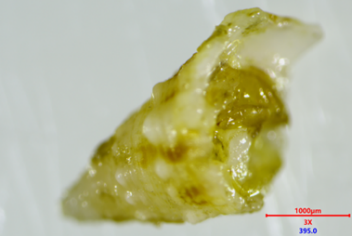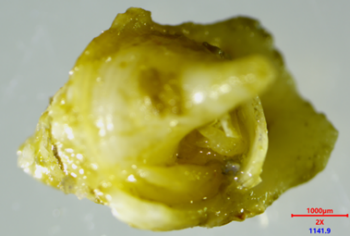  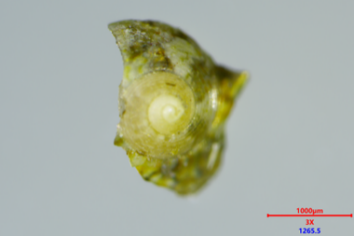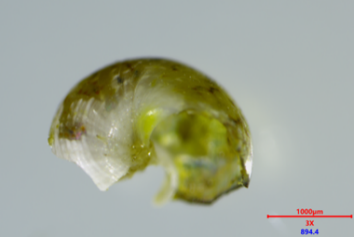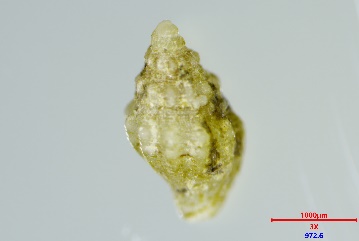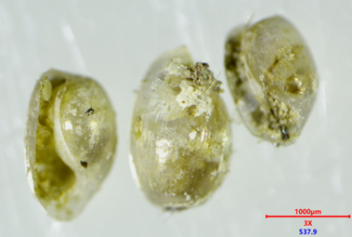  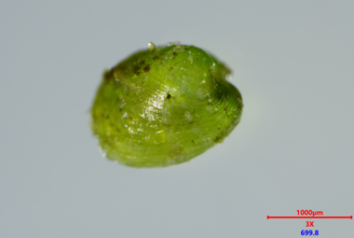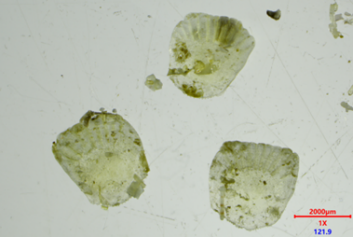  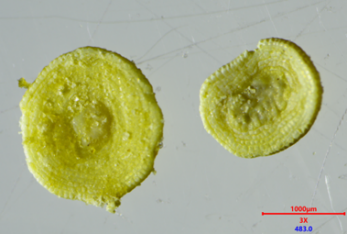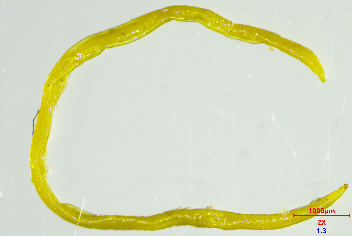 |
| *L.kasmira* | Crabs, Fish | 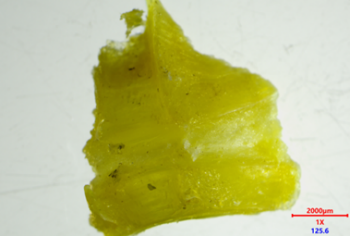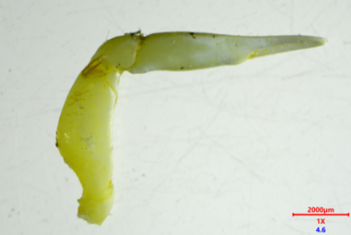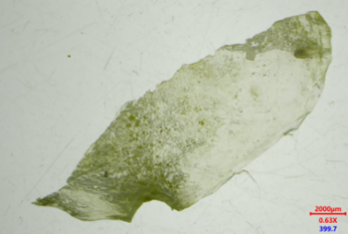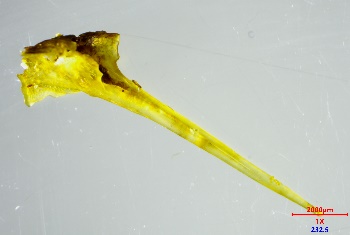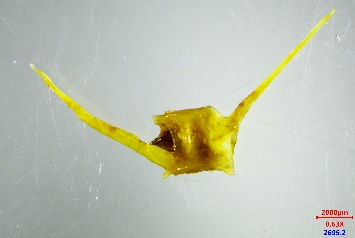 |
| *C.urodeta* | Crabs, Fish | 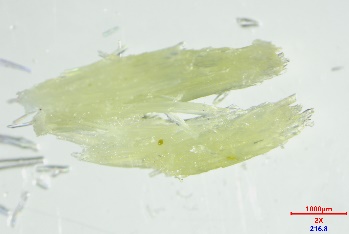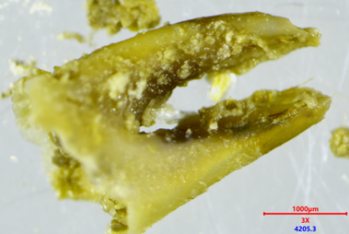 |
| *E.hexagonatus* | Fish, Shrimp | 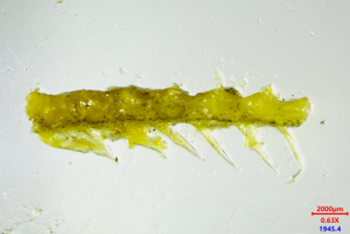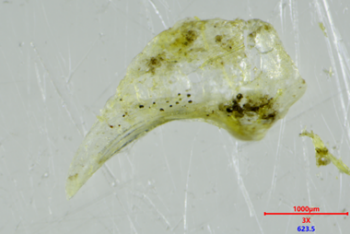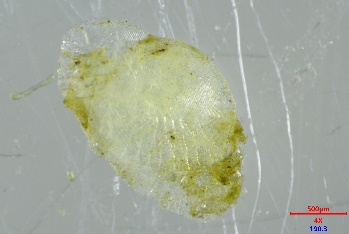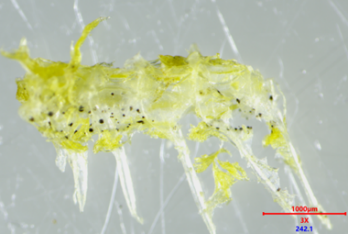  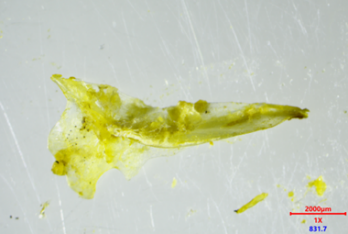 |
